# District nurses’ experiences with involuntary treatment in dementia care at home: a qualitative descriptive study

**DOI:** 10.1186/s12912-023-01553-w

**Published:** 2023-10-19

**Authors:** Vincent R.A. Moermans, Jan P.H. Hamers, Hilde Verbeek, Bernadette Dierckx de Casterlé, Koen Milisen, Michel H.C. Bleijlevens

**Affiliations:** 1 Department of Health Services Research, Care and Public Health Research Institute, Faculty of Health Medicine and Life Sciences, Maastricht University, Duboisdomein 30, Maastricht, 6229 GT The Netherlands; 2 Department of Nursing, White Yellow Cross Limburg, Genk, Belgium; 3 Living Lab in Ageing and Long-Term Care, Maastricht, the Netherlands; 4 Department of Public Health and Primary Care, Academic Centre for Nursing and Midwifery, KU Leuven, Leuven, Belgium; 5 Department of Geriatric Medicine, University Hospitals Leuven, Leuven, Belgium

**Keywords:** Nurses, Involuntary treatment, Dementia, Home care, Qualitative research

## Abstract

**Background:**

Research shows that half of person(s) living with dementia (PLWD) receive care which they resist and/or have not given consent to, defined as involuntary treatment. District nurses play a key role in providing this care. Knowledge about how district nurses experience involuntary treatment is lacking. Therefore, the aim of this study was to describe the experiences of district nurses who used involuntary treatment for PLWD at home.

**Methods:**

A qualitative descriptive design using semi-structured interviews. Sixteen district nurses with experience in involuntary treatment for PLWD were recruited through purposive sampling. Data were analysed using the Qualitative Analysis Guide of Leuven.

**Results:**

District nurses’ experiences with involuntary treatment were influenced by their involvement in the decision-making process. When they were involved, they considered involuntary treatment use to be appropriate care. However, at the moment that involuntary treatment use was started, district nurses were worried that its use was unjust since they wished to respect the wishes of the PLWD. Eventually, district nurses found, from a professional perspective, that involuntary treatment use was necessary, and that safety outweighed the autonomy of the PLWD. District nurses experienced dealing with this dilemma as stressful, due to conflicting values. If district nurses were not involved in the decision-making process regarding the use of involuntary treatment, family caregivers generally decided on its use. Often, district nurses perceived this request as inappropriate dementia care and they first tried to create a dialogue with the family caregivers to reach a compromise. However, in most cases, family caregivers stood by their request and the district nurse still provided involuntary treatment and found this difficult to tolerate.

**Conclusions:**

Our results show that district nurses experience involuntary treatment use as stressful due to dealing with obverse values of safety versus autonomy. To prevent involuntary treatment use and obverse values, we need to increase their ethical awareness, communication skills, knowledge and skills with person-centred care so they can deal with situations that can evolve into involuntary treatment use in a person-centred manner.

## Introduction

Person(s) living with dementia (PLWD) wish to age in place and have a voice in their care [1]. Person-centred care (PCC) is a fundamental principle in providing high-quality dementia care at home [2, 3]. PLWD have the right to receive person-centred, coordinated and quality care throughout their illness [4]. PCC involves meeting the needs and preferences of PLWD, and taking into consideration the needs, goals and abilities of all caregivers involved. However, providing PCC in dementia care at home is confronted with several barriers related to the caregiver such as lack of practical and emotional support and lack of knowledge about PCC and attitudes about dementia [5, 6]. When dementia evolves further, PLWD experience problems with expressing their wishes, and eventually they may lose (part of) their decision-making capacity [6]. Thus, caregivers often decide what care is in the best interest of PLWD [7, 8]. According to the ethical code of nurses, it is important that, in these situations, nurses be their patients’ advocates and assist the PLWD in the decisions made in order to deliver person-centred and dignified care [9]. When a PLWD does not agree with the provided care, this can lead to agitation and/or resistance to the care. This can be distressing for the PLWD, their family caregivers and the nursing staff [7, 10–12].

### Background

Several terms are used in the literature to describe the care that persons resist or do not provide consent for, such as coercive care, resistiveness to care, refusal of care, forced treatment and involuntary treatment [8, 13–15]. This study uses the term ‘involuntary treatment’, which is defined as care provided without the consent of the person receiving it and/or to which this person resists, including the use of physical restraints, psychotropic medication and non-consensual care [13]. Recent research shows that involuntary treatment is provided to half of the PLWD receiving professional home care in Belgium and the Netherlands [12]. In Western countries, the presence of known risk factors for involuntary treatment use, such as caregiver burden, living alone, greater activities of daily living (ADL) dependency and poorer cognitive ability, are increasing due to demographic and socio-economic evolutions [12, 16]. Family caregivers and district nurses play a key role in the decision-making process regarding the use of involuntary treatment [7, 12]. District nurses perceive involuntary treatment as a regular part of nursing care, having neither a positive nor negative attitude towards its appropriateness [17]. Since involuntary treatment is in conflict with person-centred dementia care and ethics of nursing, and more person-centred alternatives exist, involuntary treatment needs to be prevented [2, 4, 5, 9].

If we wish to prevent involuntary treatment, insight is needed into caregivers’ experiences regarding the decision-making process and its application. Recently, several studies have been published on family caregivers’ experiences regarding measures defined as involuntary treatment [7, 18–20]. Family caregivers consider safety and autonomy as important values. However, they struggle with finding the right balance between them and experience dealing with these dilemmas as stressful [7, 18]. They apply several strategies to deal with the resistance towards their care and the creation of a safe environment [18–20]. Recently, a study was published concerning how district nurses experience and encounter resistance to care from PLWD [21]. This study showed that district nurses tried to avoid forced treatment and to provide adapted care to PLWD who resisted care. However, little is known about the experiences and decision-making processes of (district) nurses, when involuntary treatment was actually applied in dementia care at home. Therefore, insight is needed into how district nurses perceive involuntary treatment usage and how they deal with care situations in which involuntary treatment is used. Based on these insights, person-centred interventions can be developed for district nurses in order to prevent involuntary treatment use.

This study focuses on involuntary treatment use among PLWD at home. Therefore, the research questions are:

- What are the experiences of district nurses regarding the application of involuntary treatment use?
- To what extent are district nurses involved in the decision-making processes concerning involuntary treatment usage?

## Methods

### Design

A qualitative descriptive approach was adopted based on naturalistic inquiry to gain a straight and rich description of the experiences of district nurses regarding involuntary treatment usage [22]. Semi-structured interviews were conducted with district nurses in Belgium. Data were analysed using the Qualitative Analysis Guide of Leuven (QUGOL), a method that is inspired by the constant comparative method of the Grounded Theory Approach [23]. To ensure rigour, we followed the “Consolidated criteria for reporting qualitative research (COREQ)” guidelines [24].

### Setting

Participants were district nurses from an organisation that provided professional at-home nursing care in the eastern part of Belgium. They administered nursing care at home and paid attention to family and social circumstances. District nurses were responsible for planning, coordinating, performing and evaluating the nursing care provided in a patient’s home environment and who belonged to their district [25]. They provided this care together with family caregivers and other professional caregivers like general practitioners (GP) [25]. Every district had a team of nurses, comprising a responsible district nurse, assisted by permanent district nurses to ensure 24/7 care continuity. This meant that several nurses provided care for one PLWD. To ensure continuity and uniformity of the given care, these nurses communicated with each other through an online electronic patient record.

### Sampling

Maximum variation sampling was used to create a diverse sample of participants (having few or many years’ experience as a district nurse, of young and older ages, male and female nurses, having a lower or higher educational background, perceiving caring for PLWD as burdensome or not, having an educational background in dementia care at home and involuntary treatment or not), who had experience with involuntary treatment use among PLWD in the past 12 months [26].

### Method of approach

Prior to this study, from May to June 2021, we conducted a cross-sectional study using an online survey tool among 296 district nurses to explore their attitudes towards the use of involuntary treatment and their opinions about the restrictiveness and discomfort of involuntary treatment measures in dementia care at home [17]. At the end of this online survey, information was given about the researchers, aim, method and context of the current study. If district nurses were interested, they could voluntarily apply to participate in this study by completing an online application form that requested the following information: age, years of experience as a district nurse and how many times in the past 12 months they were confronted with the use of involuntary treatment in PLWD. Sixty-one district nurses indicated that they were interested in participating in the current study and 51 district nurses met the inclusion criterion, namely having experience with involuntary treatment use in the past 12 months. Using maximum variation sampling, 16 district nurses were selected for interview. Table 1 provides an overview of the district nurses’ characteristics. The researcher (V.M.) contacted the selected district nurses by phone to inform them about the study and plan an appointment to conduct the interview. Participation was entirely voluntary and participants were free to withdraw at any time. None of the participants dropped out during the study. All district nurses received written and verbal information about the study in advance.

**Table 1:** Characteristics of district nurses (N = 16)

	Number
**Age**	
20–29 years	2
30–39 years	8
40–49 years	2
50–59 years	4
**Years of experience as a district nurse**	
0–1 years	2
2–5 years	3
6–10 years	2
11–20 years	4
21–30 years	2
More than 30 years	3
**Gender**	
Male	1
Female	15
**Educational background**	
Diploma degree	7
Bachelor’s/Master’s degree	9
**Did the participant receive an education in dementia care at home**	
Yes	9
No	7
**Did the participant receive an education in involuntary treatment use**	
Yes	6
No	10
**Perceived burden of caring for persons living with dementia**	
Seldom	2
Now and then	11
Often	3

### Data collection

In October 2021, all interviews were conducted by the researcher (V.M.) at the participants’ work office. Only the participant and the researcher (V.M.) were present during the interviews. The interviewer (V.M.) was a male PhD student, who also worked as a staff member in the organisation where the participants were employed. However, he had no direct relationship with the participants, at the time of the interviews. The interviewer had a background in district nursing, experience in dementia care, involuntary treatment, conducting and analysing qualitative research. A literature review [7, 13, 17, 27, 28] and two pilot interviews guided the development of the interview guide, which was further revised in response to emerging insights and discussions within the research team. Informed consent was obtained from each participant before the start of their interview. All interviews were conducted in Dutch, audio-recorded with the participants’ permission and transcribed by the researchers. It was anticipated that interviews would last approximately 45 min. Only the principal researcher knew the participants’ identities. Data from participants were anonymised after transcription and treated confidentially. The interviews were performed using an informal interview technique including an open and broad conversation focusing on the participant’s experiences. First, the interviewer explained what involuntary treatment use is to the participant. Then, the interviewer asked the participant to briefly describe some situations of involuntary treatment use among PLWD at home in which they was involved as a district nurse. Next, the participant was asked to describe one of these care situations in detail. Subsequently, the interviewer asked spontaneous follow-up questions, based on the interview guide (see Table 2). After 16 interviews (describing 34 cases of involuntary treatment use among PLWD) were conducted, the results were discussed with the research team. They concluded that data saturation had been reached, as the last four interviews confirmed the themes previously found without introducing new or additional themes or information.

**Table 2:** Interview guide

Main question:
Can you describe to me which kind of involuntary treatment use in dementia care at home you’re mostly involved in as a district nurse?
Can you describe to me in detail one of these care situations that you just mentioned?
Based on the information provided, the following follow-up questions were asked:
1) Regarding the use of involuntary treatment
• What just happened?
• Which events led to the use of involuntary treatment? What preceded it?
• How did you deal with this?
2) Decision-making process and actors involved
• Who made the decision to use involuntary treatment?
• How was the decision to use involuntary treatment made?
• Who was involved in the decision-making process?
• What was everyone’s role in the decision-making process?
• What was your role?
• What was the role of the other nurses and professional caregivers?
• How did the other nurses and healthcare professionals deal with the decision to use involuntary treatment?
• How was the PLWD and/or their representative involved in this decision?
3) Feelings of the caregiver
• How did you experience the use of involuntary treatment with this patient?
• What did you think when you first used involuntary treatment for this patient?
• How do you feel now about the use of involuntary treatment with this patient?
• What influence has this care situation had on you as a person?
• How did the patient and their loved ones experience the use of involuntary treatment? And to what extent has this influenced your actions?
4) (Experienced) support
• What support did you have in dealing with this care situation?
• How did you experience this support?
• Who helped you the most in dealing with this care situation?
• How did you experience the support from the organisation?
• What helps you the most in dealing with such care situations?
• What did you miss concerning being able to provide good care in this situation?
• Can you briefly describe what good care means to you?
5) Closing question
• If you look back on this care situation, how would you have dealt with it now?
• What did you learn from this care situation?
• What advice would you give to new employees to deal with such situations?

Depending on the remaining time, the interviewer will ask for another situation to be described. The interviewer takes into account that the interview will not last longer than 45 minutes.

### Data analysis

Data analysis was based on the Qualitative Analysis Guide of Leuven (QUAGOL), an iterative guidance tool for qualitative data analysis that consists of a preparatory coding process and an actual coding process [23]. During the preparatory part, four researchers (V.M., M.B. and two research assistants) applied a case-oriented approach that stimulated them to first analyse and understand each case as a whole. They individually read the transcripts and developed a list of preliminary themes. Similarities, differences and connections among different themes within and across interview schemes were discussed by the four researchers. By discussing the different themes, they gradually identified common themes within and across the interviews, which resulted in a final list of themes for the actual coding procedure using qualitative software (Maxqdata 2022®). One researcher (V.M.) performed the actual coding process. The coding process was guided by a list of codes that organised the themes within a tree structure with different levels. First, all data were coded by linking each fragment of text to one of the themes from the list. Then, the usability of the codes and themes were discussed by the four researchers. In the following step, two researchers (V.M., M.B.) individually distilled the storyline from the findings and themes. These findings were discussed and submitted to the research team (V.M., M.B., H.V., B.D.d.C., K.M., J.H.) until consensus was reached.

### Rigour/trustworthiness

The study’s trustworthiness was examined in terms of credibility, dependability, confirmability and transferability as described by Lincoln and Guba [29, 30]. For credibility, the analysis process was peer reviewed (i.e. frequently reviewed within the research team to establish uniformity in themes and relationships and to encourage the researchers’ reflexivity. The research team was systematically and continually encouraged to be attentive to the context of knowledge development and, more specifically, to their own impacts on the collection, analysis, and interpretation of data) In addition, the results were peer debriefed (i.e. results were discussed with five district nurses who specialised in dementia care at home and who acknowledged the findings of this study. These five district nurses did not belong to the group of nurses interviewed). Concerning dependability, we maintained a detailed audit trail (e.g. audio files, interview transcripts, field notes, notes of the preparation of the coding process, list of contextual and analytical themes and description of themes). Additionally, we conducted researcher triangulation (i.e. four members of the research team held discussions throughout the data analytic process to ensure the selection of consistent themes). To ensure confirmability, we provided thick descriptions (i.e. relevant citations to illustrate the generated themes) and performed member checking by summarising participants’ responses at the end of each interview. Finally, to guarantee transferability, thorough descriptions of the research setting, characteristics of the participants, applied measures and processes were provided.

## Results

**Fig. 1:**
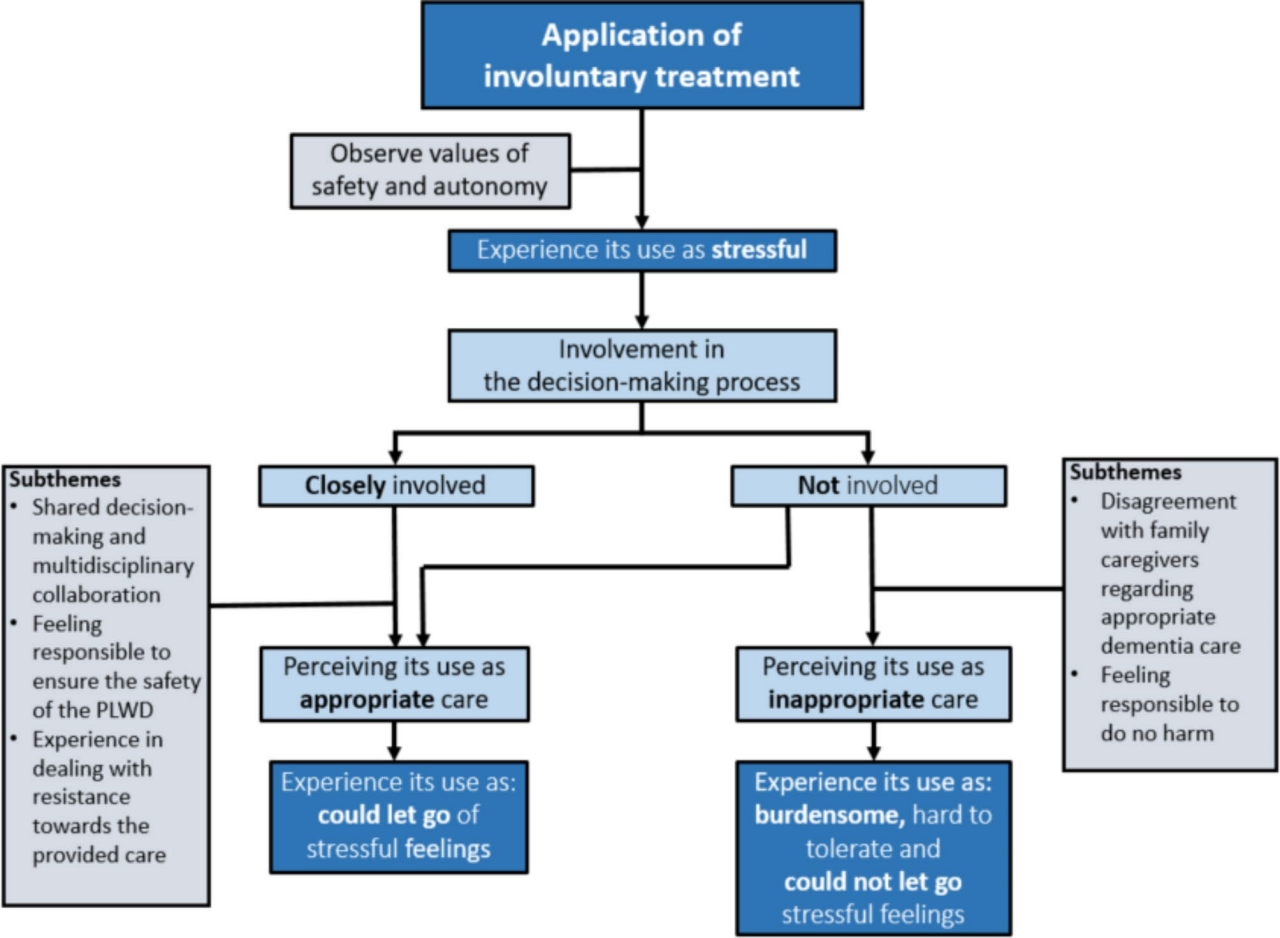
District nurses’ experiences with involuntary treatment in dementia care at home

Figure 1 illustrates that district nurses’ experiences with involuntary treatment usage depended on the extent to which they were involved in the decision-making process. Table 3 shows the district nurses’ experiences with the use of involuntary treatment. When they were involved, they considered involuntary treatment use to be appropriate care. Initially, they were worried that involuntary treatment was unjust since they wished to respect the wishes of the PLWD. However, after a while, district nurses found, from a professional perspective, that involuntary treatment use was necessary and that safety outweighed the autonomy of the PLWD. If district nurses were not involved in the decision-making process regarding the use of involuntary treatment, family caregivers usually decided on its use. Often, district nurses perceived this request as inappropriate dementia care and they first tried to create a dialogue with family caregivers to reach a compromise. However, in most cases, family caregivers stood by their request and the district nurse still provided involuntary treatment and found this difficult to tolerate.

**Table 3:** Applied measures of involuntary treatment

	Closely involved in the decision made	Not Involved in decision made		Total
	Perceived as appropriate care	Perceived as appropriate care	Perceived as inappropriate care	
Non-consensual care				
1. Forced hygiene	10	6	8	24
2. Hiding and administration of medication	2	2	4	8
3. Shutting off gas, water and/or electricity			1	1
4. Restriction of fluids			1	1
Psychotropic medication				
5. Use of sedatives	5	1	3	9
6. Use of anti-psychotics	1		1	2
Physical restraints				
7. Bilateral bedrails	6		6	12
8. Locking in house		1	4	5
9. Camera surveillance	1		2	3
10. (Wheel)chair with locked tray table	2			2
11. Fixation belt	1			1
12. Gloves			1	1
13. Sleep suit	1			1
14. Special sheet			1	1
15. Fixating arms and hands during care	1			1

Note: In two thirds of the cases, multiple measures were applied

### Experiences of district nurses closely involved in the decision-making process

In 14 of the 34 described cases, involuntary treatment use was a deliberate and shared decision between district nurses, family caregivers and/or general practitioner. In their experience, involuntary treatment was mostly stressful due to opposing feelings. If they were first confronted with resistance, they were in most cases worried that they were providing care that was unjust because they wished to respect the autonomy and dignity of the PLWD. However, after a while, they indicated that, from a professional perspective, the safety of the PLWD outweighed respecting their wishes. Therefore, in all the discussed cases, they perceived involuntary treatment as appropriate care and could justify for themselves the necessity of involuntary treatment use and let go of their mixed feelings regarding safety versus autonomy. These experiences were influenced by: (a) shared decision-making and multidisciplinary collaboration; (b) their sense of responsibility to ensure the safety of the PLWD; and/or (c) their experiences in dealing with involuntary treatment use:

I think respecting a patient’s wishes is an instinctive feeling for me. I always think, you do nursing with your heart, and only if you do that, can you be a good nurse. But, at that moment [applying involuntary treatment] I know in my head that it has to be done for the safety of the patient, but in my heart, it hurts. It’s a mixed feeling. (33-year-old district nurse with 12 years’ experience)

I find it very difficult to force medication. On the one hand, I think it’s important to respect the wishes of the PLWD, but on the other hand, I think it’s important that the patient takes his medication for his health. Because imagine if he doesn’t take it. It’s double. (36-year-old district nurse with 4 years’ experience)

#### Shared decision-making and multidisciplinary collaboration

When the PLWD came into care, they mostly agreed with the provided care such as being washed by district nurses or the administration of medication. A nursing care plan was drawn up by the district team for each patient that came into care. This nursing care plan was accessible for every caregiver that has a therapeutic relationship with the PLWD. However, the district team were not always aware of the treatment plan of the other involved healthcare providers such as the general practitioner (GP), specialist physician or psychologist.

When the functional and cognitive capabilities of the PLWD declined and district nurses determined that more nursing care was needed (hygienic care for example due to self-care deficiency or to prevent family caregiver burden), they almost always discussed this bilaterally with one of the members of the multidisciplinary team of the PLWD and an agreement was reached to apply involuntary treatment. In most cases this was a family member and the rest of the team was then informed about the decision made. These decisions were, in most cases, practical, effective and short-term solutions, based on former experiences of the involved caregivers like forced hygienic care or the use of physical restraints. Almost always, the multidisciplinary team of the PLWD consisted of the district nurse, family and/or GP, who met with each other if one of the members deemed it necessary. However, the PLWD was mostly not involved in these decisions. In these cases, the PLWD often started to openly question and/or oppose the necessity of the care received. District nurses found this to be stressful to deal with because they did not expect it, understand the behaviour of the PLWD, did not know how to react to the resistance and/or deal with it. In general, they indicated that they found it difficult to connect with the PLWD and to gain insights into why the PLWD rejected their care, which was most often due to insufficient verbal skills of the PLWD:

At the request of the son, we started administering the medication once a week. In the beginning it went well, and she needed a little support from us. Then after a year, we saw that she (PLWD) had more problems with her medication. Also, we doubted if she washed herself regularly, because we noticed that she no longer had on clean clothes. At the start, it was very difficult to wash her, because she did not allow it. She always said, I’ve already been washed. She did not know that she had not washed herself. Together with the other involved caregivers, we tried to convince her to wash herself. Yes, I think that sometimes, someone said look, we’re going to wash you now. And that this happened under force. (42-year-old head nurse with 21 years’ experience)

In almost all discussed cases where the PLWD verbally and/or non-verbally resisted or rejected the care (e.g. shouting, swearing and/or bodily harm [e.g. passive attitude, pushing away, hitting]), district nurses often experienced this as more stressful and discussed with their colleagues, the family and GP of the PLWD how they should deal with it. They exchanged advice and a mutual agreement was reached on how to deal with this resistance:

When the PLWD resists its care, I sometimes think it’s just me, or it’s something else. That is sometimes difficult. That I don’t know if I’m doing something wrong, or if it’s up to me personally. But by reading the observations in the electronic patient record, I notice that other nurses also experience this problem. That they say this week it went well and the next day it was arduous. Then I know it has nothing to do with us. Then I can better place the behaviour of the PLWD and then I am reassured and can let go of my doubts. (45-year-old district nurse with 23 years’ experience)

Additionally, in several cases, family caregivers assisted district nurses during involuntary treatment by distracting their next of kin or clamping their arms during hygienic care. District nurses experienced this assistance as very supportive and indicated that although involuntary treatment was sometimes stressful, they experienced it as bearable:

The family involves us closely in the care and help us. We were not alone and they were not alone. When the PLWD was taken care of, I never feel like that I’m exhausted or tired. (36-year-old district nurse with 4 years’ experience)

#### Feeling responsible to ensure the safety of the PLWD

At first, most of the participants perceived involuntary treatment use as difficult. They were mostly worried that involuntary treatment was unjust because as a human they found it important to respect the autonomy and dignity of another human being and mentioned that involuntary treatment drastically restricted their freedom. On the other hand, all the district nurses found it professionally necessary to apply involuntary treatment to ensure the safety of the PLWD, protect the PLWD against wandering, incorrect medication intake, skin damage due to urine burns and/or caregiver burden:

Above all the safety of the patient comes first. Because suppose you do not physically restrain him [PLWD], to respect his self-esteem and he gets up and he falls, then yes, that’s not okay. Then the situation is worse than it already was. (45-year-old nurse with 23 years’ experience)

Furthermore, they all perceived involuntary treatment usage to be appropriate care since it was: (1) discussed by a multidisciplinary team and an agreement was reached (family, GP, fellow district nurses); (2) needed in the context of PLWD safety needs; (3) planned and delivered in a qualitative manner; and/or (4) in accordance with the personal values/norms of the nurses:

I think we were able to provide good care. We have always been able to anticipate in time. By discussing how we were going to do it. Both with the doctor, with the family, with everyone. I think we acted correctly and in a timely manner for the safety of the PLWD. (35-year-old district nurse with 12 years’ experience)

In addition, district nurses often indicated that they experienced the resistance towards the care they delivered as a behavioural symptom of dementia. However, when they were first confronted with this resistance towards their care, district nurses were confused and asked themselves whether the use of involuntary treatment was unjust. Eventually, they indicated that they could easily let go of this feeling when they went home. They mentioned that they could justify its use, as not using involuntary treatment would cause more harm to the PLWD than respecting their voice:

It still remains difficult to lock someone up, but on the other hand if you look at it professionally, you understand that it is necessary. But from a human point of view I think yes … you do deprive someone of their freedom. If you’ve been working for a while, then yes, you understand why it is done. Then you can place it better. (32-year-old district nurse with 10 years’ experience)

#### Experience dealing with resistance towards the provided care

If district nurses could not bend and/or handle the resistance to or rejection of their care, they often experienced involuntary treatment use as more stressful. Table 1 shows that two thirds of the participants had some knowledge regarding dementia care and one third had once received education regarding involuntary treatment use. These district nurses, who had several years of experience and/or who knew the PLWD well, said that they found the application of involuntary treatment in general to be less stressful because they could better anticipate and/or bend the resistance towards their care by, for example, being firm, leaving and returning later, distracting or persuading them. Early career district nurses and/or those who did not know the PLWD usually found it more difficult to deal with resistance from the PLWD and therefore, were not always able to provide the planned care because: (1) they did not know how to approach the PLWD; and/or (2) they questioned the use of involuntary treatment more. Early career nurses found the support and advice of colleagues with more experience to be very helpful:

It helps if you can rely on someone who knows the PLWD through and through and has experience. I think that’s an important point, that you just need to know how to approach someone. Because that is difficult to know in advance, because everyone differs in character. (29-year-old district nurse with 1 years’ experience)

Colleagues who have been working here for 30 years are more likely to apply involuntary care than younger colleagues. Because older colleagues just perform and ask less questions compared to younger nurses. Younger colleagues often ask the question, is it okay what we do? They will question that more. (34-year-old district nurse with 12 years’ experience)

### Experiences of district nurses that are not involved in the decision-making process

In 20 of the 34 cases described, district nurses were not involved in the decision to apply involuntary treatment. Often the decision had been made by others (e.g. family, GP and other professional caregivers) before the PLWD came into care and district nurses were requested to provide involuntary treatment and did so. In seven of the 20 cases described, district nurses agreed with the family that involuntary treatment was necessary and that it was appropriate care. In these cases, they experienced involuntary treatment as described above. However, in 13 of the 20 cases, district nurses found the request for involuntary treatment to be inappropriate dementia care and experienced its use as burdensome and struggled with providing it. In these cases, district nurses often first tried to create a dialogue with the PLWD’s family to reach a compromise regarding appropriate dementia care. In most cases, however, they said that the family stood by their request. In these situations, district nurses found the use of involuntary treatment difficult to tolerate. This experience was influenced by: (a) disagreement with family caregivers regarding appropriate dementia care; and (b) their responsibility to do no harm:

I find it difficult when a PLWD has to stay in bed. Because it’s too dangerous, according to others. While the PLWD says he wants out. I have a hard time with that, it conflicts with my values and norms because those people can also sit up. This I struggle with. (35-year-old district nurse with 12 years’ experience)

#### Disagreement with family caregivers regarding appropriate dementia care

If district nurses disagreed with the family caregivers about the use of involuntary treatment, this was mainly because they perceived their requests as too far-reaching and to be irresponsible care and therefore, experienced it as inappropriate. This mostly involved the use of physical restraints like locking the PLWD in the house or bilateral bedrails. Moreover, when it involved forced hygienic care, district nurses described that they were not aware that the PLWD would resist or reject their hygienic care when they started with the care. When they were confronted with resistance to their care, they usually tried first to provide it. They mentioned that the family expected that they took up their responsibility as a nurse and administer the required hygienic care or locking the PLWD up in their own house. Additionally, they wanted to meet this expectation. However, they usually perceived the use of physical restraints as more far-reaching than the use of non-consensual care. Subsequently, they tried to be an advocate of the PLWD by creating a dialogue with the family to discuss their request and reach a compromise regarding appropriate dementia care:

Because Mrs. [PLWD] says no, I’d rather not have that bilateral bedrails raised. But, the daughter says, that they must be raised. While you have people with dementia, who are still okay, where there is no danger. Who still have bright moments. You listen to both the family and the patient. And you try to find a compromise. I asked myself, when are you doing well, sometimes you don’t know it. (38-year-old district nurse with 1 years’ experience)

However, compromise was often not reached; the family stuck to their decision and the district nurse provided the requested care. In addition, district nurses indicated that in those cases, there was often a lack of support and/or mutual agreement between the nursing staff and the family. In these situations, they referred to the family’s decision to justify why involuntary treatment was used:

It is different if you are a district nurse or a hospital nurse. You enter someone else’s house and therefore you cannot set your own rules and laws. You can only try to enter into a dialogue, but if the family says that’s the rule, then that’s the rule. (27-year-old district nurse with 6 years’ experience)

The decision to close the gate was a decision of the children, for safety reasons. In our opinion, the children did not know that alternatives were available. But for us, it was especially difficult that we as nurses were expected to carry out the requested care. While no one in the team felt comfortable with providing that care. We were not involved in the decision-making process, we were instructed. (34-year-old district nurse with 12 years’ experience)

In all these cases, district nurses described involuntary treatment use as burdensome and hard to tolerate. By discussing these situations within the nursing staff, they were able to vent and usually, they could gradually accept the decisions made, although they still had reservations about them:

That [locking up the PLWD] was discussed a number of times, during team meetings. In the beginning, this was discussed very frequently, but after a while, we resigned ourselves to it. (54-year-old head nurse with 33 years’ experience)

#### Feeling responsible to do no harm

If the district nurse felt that by providing the requested involuntary treatment there was a potential risk that the PLWD could have an accident and be injured, they found it very hard to apply. Usually this involved locking the PLWD in their home or using bilateral bedrails. District nurses were worried that the PLWD could die of suffocation or burns since they could not leave their home or bed. Because district nurses stated that they felt responsible for the safety of the PLWD and to do no harm and they also realised that they could be held liable and even be blamed for something that they did not want to do, they struggled with their feelings and could not let go of it when they went home:

I find it difficult to lock someone up in the house because if a fire breaks out, those people are locked up there. Should something happen, I was the last person to see that person and I made sure he couldn’t go outside. Then it was me. Then it was my responsibility and that rankles with me. (53-year-old district nurse with 33 years’ experience)

## Discussion

The results from this study indicate that many district nurses found the application of involuntary treatment stressful and had dilemmas when applying it, especially in the beginning or when they felt it might do more harm than good. District nurses’ experiences depended on their involvement in the decision-making process. When they were involved, they considered involuntary treatment use to be appropriate care despite mixed feelings and perceived it as stressful. Initially, they were worried that involuntary treatment was unjust since they wished to respect the wishes of the PLWD. However, eventually they found professionally that involuntary treatment use was necessary and that safety outweighed the autonomy of the PLWD. If district nurses were not involved in the decision-making process, they considered the request for involuntary treatment inappropriate dementia care. However, they still provided it and experienced its use as burdensome and struggled with it.

Our results suggest that when district nurses were confronted with involuntary treatment they experienced this as stressful due to cognitive dissonance, as they experienced obverse feelings regarding autonomy and safety [10]. Cognitive dissonance is a phenomenon that arises when persons experience psychological discomfort when they are trying to meet two or more opposing demands at the same time or engage in activities that conflict with their beliefs or values. When persons experience these obverse cognitions, they perceive it as psychologically uncomfortable, and wish to reduce this dissonance by rationalising their actions [10, 31]. We found indications that district nurses did this by: (1) changing their cognition regarding involuntary treatment by perceiving it as appropriate dementia care by referring to the clinical picture of dementia, medical knowledge or personal values; (2) managing of refusals of care (e.g. leaving and returning later, changing the right moment of care, being firm, bringing in others, distracting or persuading them; or (3) creating new consonant cognitions by finding that providing safe care was more important than respecting the voice of the PLWD [20, 31]. The finding that safety outweighed the autonomy of the PLWD showed that the normative arguments district nurses used to decide which care was needed were generally based on a biomedical ethical approach [32]. Since they mainly focused on the bodily needs of the PLWD like protecting against harm and less on their moral needs like involving them in decisions about their care, making the principles of non-maleficence (e.g. protection from harm) and beneficence (e.g. enhancing the safety or personal well-being of the PLWD and/or family) leading in their normative arguments. Further, district nurses were not always sufficiently aware that the resistance during their care could be a signal of the bodily autonomy of the PLWD to indicate that they did not agree with how their care was provided and/or it did not correspond to their habits and needs [33]. When district nurses used a biomedical approach rather than a biopsychosocial one like PCC, this increased the risk that routine care like hygienic care takes priority over psychosocial aspects of the care provided like respecting the bodily autonomy of the PLWD. Consequently, district nurses were hindered from observing the behaviour of the PLWD and how they responded to their care. As a result, district nurses were unaware that, for example, non-consensual care can have serious consequences on the social, psychosocial and moral well-being of the PLWD. These results indicate that if we want to provide dignity-enhancing dementia care and prevent cognitive dissonance, it is important that district nurses are more aware of the bodily signals of autonomy and discuss and evaluate their care with the PLWD [34]. These findings and the fact that several participants were not educated in dementia care and/or involuntary treatment usage, underpins the necessity that more education and training in this is needed. Therefore, health care organisations and nurse education curricula need to focus more on increasing the ethical awareness and knowledge of nurses regarding the negative consequences of involuntary treatment and support them in recognising the moral needs of the PLWD and maintaining their selfhood at home [2]. Further, to reduce the risk for cognitive dissonance and/or alleviated it, district nurses need to be trained and provided with continuous support regarding alternatives for involuntary treatment (e.g. negotiation, preventing sensory over load or under stimulation) [35–37], interactions and communications with the PLWD, approaching the PLWD during hygienic care, ability-focused approaches, distraction approach, and knowledge about PCC [38–44]. As a result, district nurses will gain more insights and skills to approach a PLWD in a more person-centred manner and to align their values and actions with each other In addition, since it was not always clear to the nursing staff how to react when confronted with involuntary treatment and/or doubted whether they had acted correctly, nursing management need to develop and provide clear written guidelines and targeted intervention strategies on how to deal with situations concerning involuntary treatment [35, 45].

Further, our results show that the multidisciplinary team of the PLWD (i.e. nurses, family caregivers and/or GPs) used a rather intuitive or heuristic decision-making process when confronted with stressful dilemmas regarding safety and autonomy, as their decisions and applied solutions in most cases were based on their own personal experiences and/or perceptions. Heuristic decision-making is optimal for simple, routine and low impact tasks to reduce the cognitive load of thought processes associated with complex and analytical thinking, and to guide decisions which are perceived as most efficient. However, when confronted with complex dilemmas regarding involuntary treatment, a rather analytical reasoning is needed, which requires evidence-based reasoning [46]. To reduce the risk of heuristic decision-making, the multidisciplinary team needs to critically reflect upon decisions to broaden their knowledge. Effective strategies for this are following a working procedure with a step by step plan, increasing the expertise of the multidisciplinary team by involving expert nurses, psychologists, advice of an ethics committee, shared decision-making and increasing knowledge regarding alternatives to involuntary treatment [46, 47].

In addition, the findings that district nurses with more years’ experience found it easier to deal with and/or bend the resistance of the PLWD and apply involuntary treatment compared to starting nurses suggest that, due to long-term exposure to stressful situations, district nurses could become desensitised or passive towards the negative consequences of involuntary treatment use and therefore, more accepting of it [10, 48].

Our findings highlight and confirm the dominant role of family in the decision-making process regarding involuntary treatment like physical restraints and that nurses mostly provided the requested involuntary treatment, although they often found it inappropriate care; also shown in earlier studies in home care and acute and residential settings [7, 12, 28, 49]. Earlier studies indicate that family caregivers often have insufficient knowledge and skills to deal properly with dilemmas regarding safety and autonomy in a person-centred manner, due to insufficient emotional support in the decision-making process from professional caregivers. This results in them relying on previous experiences, knowledge of alternatives and the practical assistance and support from family, friends and caregiver support groups [1, 7]. In addition, often, district nurses could not convince the family to change their opinion about the requested care. Eventually, they put their own professional opinion aside and provided the requested care due to a conventional way of reasoning, as was found in other studies [49, 50].

Based on the insights of this study, we formulated the following recommendations for practice, research and education. For practice: firstly, home care organisations need to foster communication skills and knowledge about PCC of district nurses so that they can successfully discuss requests regarding involuntary treatment in a person-centred manner [5, 51]. Secondly, a multidisciplinary team (general practitioner, family) must be timely in discussing decisions regarding involuntary treatment. District nurses must have a pivotal role in these discussions as the patient advocate by providing person-centred alternatives for the requested involuntary treatment. Thirdly, professional caregivers need to support family members of the PLWD in dealing with situations that can lead to involuntary treatment use in a timelier and more PCC-manner. District nurses can support family caregivers in this by discussing alternatives of involuntary treatment usage [35–37] and the underlying factors of involuntary treatment with the family caregivers of the PLWD such as caregiver burden, lack of knowledge, skills and support. Fourthly, increasing the awareness of family caregivers about caregiver burden, behavioural problems, discussing alternatives of involuntary treatment and strengthening their social network is also required [52]. Worldwide, several studies regarding multicomponent combined support programmes for the PLWD and their caregivers have been shown to be effective in emotionally and socially supporting them both [53]. With regard to research, first of all, more research is needed into how the insights and previous recommendations of this study can be integrated into existing multicomponent programmes to increase their effectiveness, in order to prevent involuntary treatment in home and residential care [38, 45, 54–58]. Secondly, our results underpin the need for studies to be conducted in order to explore possible strategies that district nurses can use to reduce the risk of cognitive dissonance and/or moral distress in a person-centred manner when confronted with involuntary treatment. Further, this study points out that interventions should be developed aimed at district nurses on order to increase their awareness, knowledge and skills regarding supporting PLWD with a diminishing decision capacity and assist them in the decisions concerning their care, in order to be their patient advocate. Finally, for education, we recommend that nurse education curricula, make it a priority to strengthen the critical ethical reflection and dialogue skills of nursing students, in order to engage in dialogue about involuntary treatment.

### Methodological considerations

Some limitations of this study must be considered. First, a limitation is the transferability of this study to other nursing settings because all participants were district nurses that worked in a professional home nursing organisation [29]. However, thick descriptions, characteristics of the participants, applied measures and processes were provided, in a way that other researchers and caregivers can assess if the findings and recommendations of this research provide valid information for their own settings. In addition, we found similar results in international studies regarding physical restraints in psychiatric care, nursing homes and hospital units [49, 50, 59]. Therefore, we believe that our findings can be transferable to other healthcare workers and settings. Second, sampling bias can be considered a limitation because we only interviewed nurses who volunteered to participate in this research, so we could have missed district nurses that had different experiences (e.g. no mixed feeling) with involuntary treatment use [60]. Thirdly, however, while several strategies were used to ensure the credibility and dependability of our results, we could not fully exclude the risk of interview bias. Interview bias (e.g. as errors by the participants, appearance or unintentional errors of interviewer) could have influenced the internal validity our study results [60]. Finally, this research only focused on the experience of district nurses. To get a thorough insight into involuntary treatment use, observations of district nurses could increase the credibility of our finding (triangulation). Further, a case-study of several ecosystems (PLWD, family, family caregivers, general practitioner, domestic carer, district nurses, etc…) regarding involuntary treatment would provide more detailed insights into its use and the decision-making process.

## Conclusion

The results from this study suggest that, depending on their involvement in the decision-making process, district nurses experienced involuntary treatment use differently. In general, they experienced its use as stressful due to cognitive dissonance regarding obverse values of safety versus autonomy. To prevent these obverse cognitions and involuntary treatment use, we need to increase district nurses’ communication skills, knowledge and skills about person-centred dementia care. Further, we need to foster ethical awareness regarding daily ethical situations of all caregivers involved in order to deal with situations that could lead to involuntary treatment use in a more PCC-manner.

## Data Availability

The datasets generated and/or analysed during the current study are not publicly available due to restrictions in the ethical approval but are available from the corresponding author upon reasonable request.

## List of abbreviations

ADL: Activities of Daily Living
e.g.: Exempli gratia – ‘for example’
GP: General Practitioner
i.e.: Id est – ‘that is’
PCC: Person-centred Care
PLWD: Persons Living With dementia
